# Supplementary material for: Global view on the metabolism of RNA poly(A) tails in yeast Saccharomyces cerevisiae
Source: Nat Commun. 2021 Aug 16;12:4951. doi: 10.1038/s41467-021-25251-w (PMC8367983; doi:10.1038/s41467-021-25251-w)
Supplement: Supplementary file 1 — Manuscript supplementary information [file 41467_2021_25251_MOESM1_ESM.pdf]

Supplemental Information

Global view on the metabolism of RNA poly(A) tails in yeast *Saccharomyces cerevisiae*

Tudek et al.

Supplemental Figures 1-6

Supplemental Tables 1-3

Supplemental References

Supplemental Figure 1

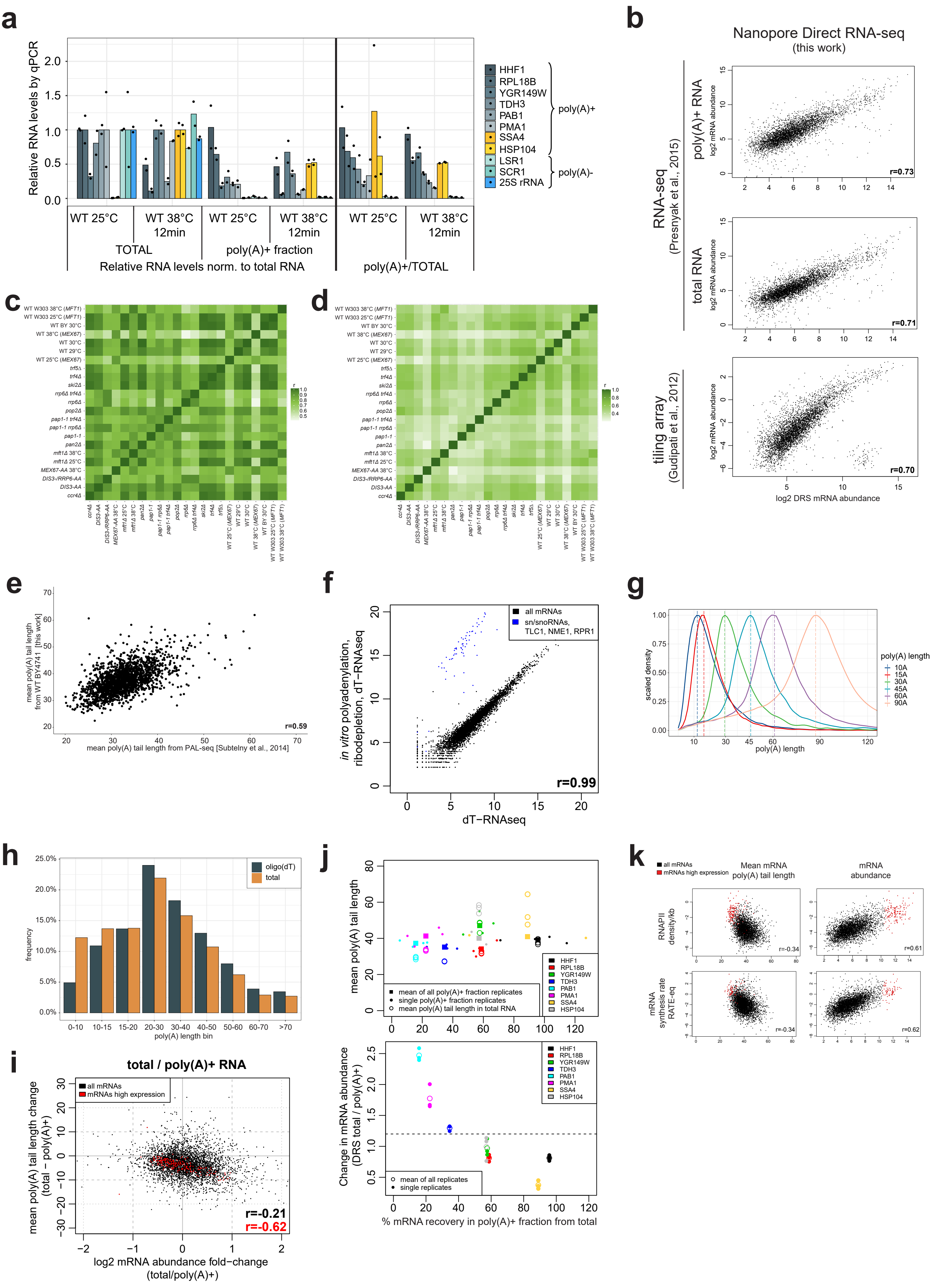

Supplemental Fig. 1 | a, Two leftmost panels, are bar plots showing mean levels of selected RNAs in the total and poly(A)$^+$ fractions determined using internally normalized reverse-transcription qPCR. The rightmost panel shows the fraction of RNA recovered from total RNA (the poly(A)$^+$ fraction divided by the total). Dots depict single data points that produced the mean. RNA was isolated from WT cells grown at steady-state at 25°C and heat-shocked for 12 min at 38°C. Tested mRNAs are specified in the key and grouped into poly(A)$^-$ ncRNAs (blue series), poly(A)$^+$ stable mRNAs (gray series), and mRNAs that were transcriptionally induced following heat-shock (yellow series). **b,** Scatterplots comparing the log2 mRNA abundance in the poly(A)$^+$ fraction DRS datasets to three published datasets:1) RNA-seq on poly(A)$^+$ fraction, 2) RNAseq after ribodepletion (both Presnyak et al., 2015[1]) and 3) tiling arrays(Gudipati et al., 2012[2]). The DRS data were prepared by averaging four wild-type DRS datasets (WT W303 grown at 25°C, two repeats of W303 grown at 30°C and WT BY grown at 30°C). The mRNAs that have altered expression in DRS compared to tiling are all intron-containing, and their abundance has been erroneously estimated due to the tiling array quantification method (Gudipati et al. 2012[2] and references therein). **c-d,** Heatmap of the correlation of DRS counts (c) and mean poly(A)-tails (d) of all datasets that were analyzed in the present study. Due to a large number of samples sequenced biological replicates were averaged to ease interpretation **e,** Scatterplot of the correlation between mean poly(A)-tail length in one of the WT BY4741 DRS datasets and PAL-seq data (Subtelny et al., 2014[3]). **e- f,** Scatterplot comparing log2 mRNA and ncRNA abundance (black and blue dots, respectively) in dT-primed libraries prepared on total RNA with or without *in vitro* polyadenylation and ribodepletion as described in Tudek et al., 2018[4] and Schmid et al., 2018[5]. **g,** Graph compares the frequency of mRNA reads in total and poly(A)$^+$ DRS containing poly(A)-tail length binned into intervals of 5-10 adenosines. **h,** Poly(A)-tail length estimation by DRS of a set of artificial spike-ins synthesized with pre-determined poly(A)-tail lengths as indicated in the

plot. **i,** Scatterplot compares on the x-axis the log2 fold change in mRNA abundance (in total compared to poly(A)$^+$ fraction) to the absolute change in mean poly(A)-tail length estimate (in total compared to poly(A)$^+$ fraction) on the y-axis. Pearson correlations are given for the entire coding transcriptome and the highly expressed mRNAs in black and red respectively. **j,** Two scatterplots showing on the x-axis the percentage of transcripts recovered from the total in the poly(A)$^+$ fraction, for arbitrarily selected mRNAs. The top panel displays on the y-axis the DRS-estimated mean poly(A)-tail in the poly(A)$^+$ and total fraction length for four wild-type strains and growth conditions (BY4741 strain grown at 30°C, and W303 strain grown at 25°C, 30°C and 38°C for 12 min.). The mean of all strains as well as single replicates are shown. The bottom panel shows on the y-axis the change in DRS transcript abundance in the total versus the poly(A)$^+$ fraction. The vertical dashed line located at 1.2 indicates the threshold used to select mRNAs with a large oligo-adenylated fraction. **k,** Scatterplot matrix of the relationship between DRS-defined mean poly(A)-tail length or log2-scaled mRNA abundance relative to log2-scaled RNAPII gene occupancy (Pelechano et al., 2010[6]) and log2-scaled mRNA synthesis rate by RATE-seq (Neymotin et al., 2014[7]). Pearson's correlation coefficients are shown on each plot.

Supplemental Figure 2

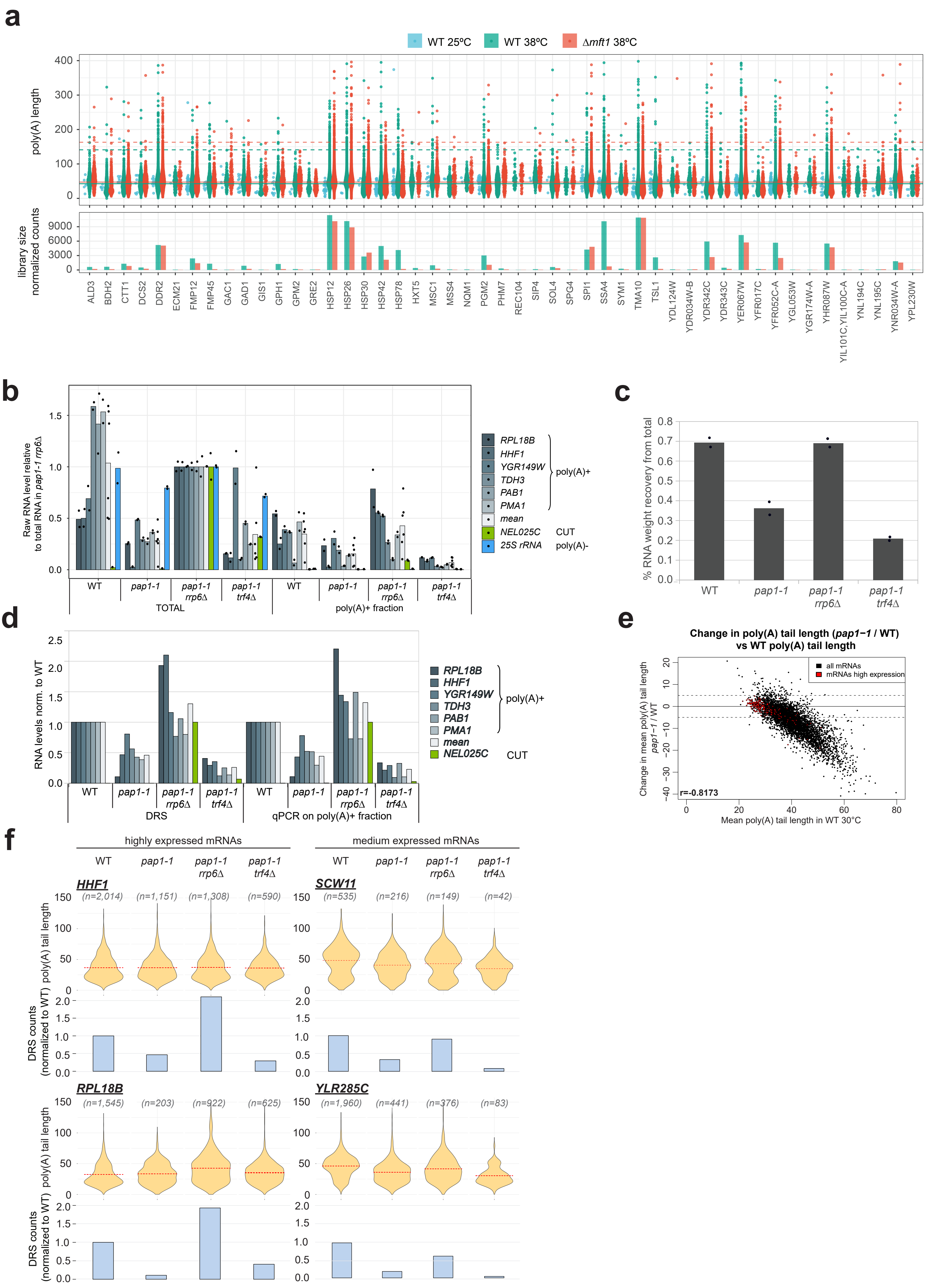

Supplemental Fig. 2 | a, The top panel shows a beeswarm plot for poly(A)-tail length distribution of a collection heat-induced transcripts in control and *mft1Δ* cells. The mean and 99.5 % quantile are marked with solid and dashed lines respectively. The bottom panel shows library size normalized counts for those mRNAs. **b,** Bar plot showing levels of six arbitrarily selected mRNAs (black and gray series), *NEL025c* CUT and *25S rRNA* in the total and poly(A)$^+$ fractions from WT, *pap1-1*, *pap1-1 rrp6Δ*, and *pap1-1 trf4Δ* cells. Mean levels of mRNAs are marked as white bars. Dots depict single data points that produced the mean. Data from reverse-transcription qPCR were normalized internally. **c,** Bar plot of the mean percentage of RNA recovered from the poly(A)$^+$ RNA fraction relative to the total as the function of its weight for WT, *pap1-1*, *pap1-1 rrp6Δ*, and *pap1-1 trf4Δ* cells. Dots depict single data points that produced the mean. **d,** Bar plot comparing library size-normalized DRS counts (in the left panel) with the levels of selected RNAs obtained from reverse-transcription qPCR in the right panel (also shown in Supplemental Fig. 2b as the poly(A)$^+$ fraction but normalized to the WT sample). **e,** Scatterplot of the relationship between WT mean poly(A)-tail length and absolute change in mean poly(A)-tail length in *pap1-1* cells compared with WT. Dashed lines mark a 5-adenosine change of mean poly(A)-tail length. **f,** poly(A)-tail length distribution of DRS reads for selected highly and moderately expressed mRNAs in WT, *pap1-1*, *pap1-1 rrp6Δ*, and *pap1-1 trf4Δ* cells and relative abundance changes for each condition (below each violin). The number of DRS reads for each transcript is indicated. Red dashed lines show the mean for each condition Bar plots below violin plots indicate the abundance of each transcript relative to the WT control.

Supplemental Figure 3

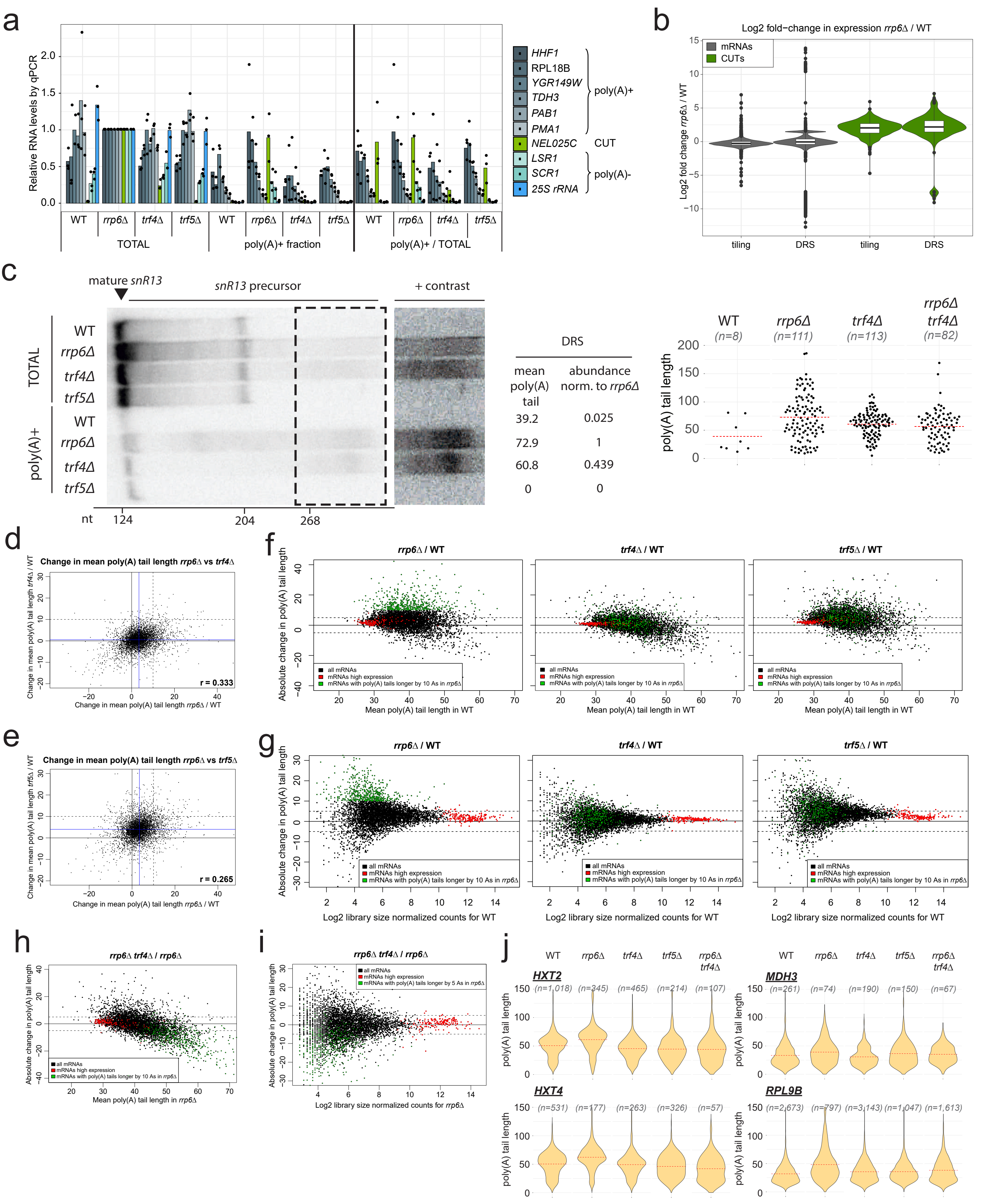

Supplemental Fig. 3 | a, Barplot that shows in the two leftmost panels internally normalized reverse-transcription qPCR-determined mean levels of selected RNAs in the total and poly(A)+ fractions for the WT, *trf5Δ*, *rrp6Δ*, *trf4Δ* strains. The rightmost panel shows the fraction of RNA recovered in the poly(A)$^+$ pool from the total. Dots depict single data points that produced the mean. As in Supplemental Fig. 1b, RNAs are grouped into poly(A)+ mRNAs as black and gray series and poly(A)- mRNAs as blue series. *NEL025c* CUT ncRNA is shown as a green series to represent exosome-dependent RNAs. **b,** Violin plot comparing fold changes in the abundance of mRNAs and CUTs in exosome mutants relative to WT between DRS datasets and tiling arrays from Gudipati et al., 2012[28]. For clarity, only CUTs that were detected in WT DRS datasets were used for the analysis. **c,** The left panel shows the Northern blot analysis of *snR13* (mature and precursor)in total and poly(A)+ fractions. The blot is representative of two biological repeats for WT and *rrp6*Δ and three for *trf4*Δ and *trf5*Δ analyzed by Northern blot. The lengths estimate is shown, based on the other mature snoRNA with well-known size (snR5, snR82, snR38), detected on the same blot. Additional blots used to produce the size marker are provided as the Source Data. The mean poly(A)-tail lengths from the DRS datasets are specified for WT, *rrp6Δ*, *trf4Δ*, and *trf5Δ* cells. The right panel shows the *snR13* poly(A)-tail length distribution of DRS reads in the form of a beeswarm plot for WT, *rrp6Δ*, trf4*Δ*, and *rrp6Δ trf4Δ* cells. Red dashed lines show the mean for each condition. In the *trf5Δ* strain, no reads were detected for *snR13*. **d,** Scatterplot of the absolute change in poly(A)-tail length for *rrp6Δ* cells compared with WT (x-axis) and an absolute change in poly(A)-tail length for *trf4Δ* cells compared with WT (y-axis). Blue lines designate mean poly(A)-tail length changes, and Pearson's correlation coefficient is given. Dashed lines show a 10-adenosine change of mean poly(A)-tail length **e,** Same as in (d), but an absolute change in poly(A)-tail length for *trf5Δ* cells compared with WT is shown on the y-axis. **f, g,** Scatterplots of the relationship between absolute change in mean mRNA poly(A)-tail length

in *rrp6Δ*, *trf4Δ*, and *trf5Δ* cells compared with WT (y-axis) and mean poly(A)-tail length in WT (f) or log2-scaled mRNA abundance in WT (g). mRNAs with poly(A)-tails that were longer by at least 10 adenosines in *rrp6Δ* are highlighted in green in the *rrp6Δ* plots. Dashed lines mark a 5-adenosine change of mean poly(A)-tail length **h, i,** Scatterplots of the relationship between absolute change in mean mRNA poly(A)-tail length in *rrp6Δ trf4Δ* compared with *rrp6Δ* (y-axis) and mean poly(A)-tail length in *rrp6Δ* (h), or log2-scaled RNA abundance in *rrp6Δ* (i). Dashed lines mark a 5-adenosine change of mean poly(A)-tail length **j,** Violin plot distributions of poly(A)-tail lengths of selected mRNAs in WT, *rrp6Δ*, *trf4Δ,* *trf5Δ*, and *rrp6Δ trf4Δ* cells.

Supplemental Figure 4

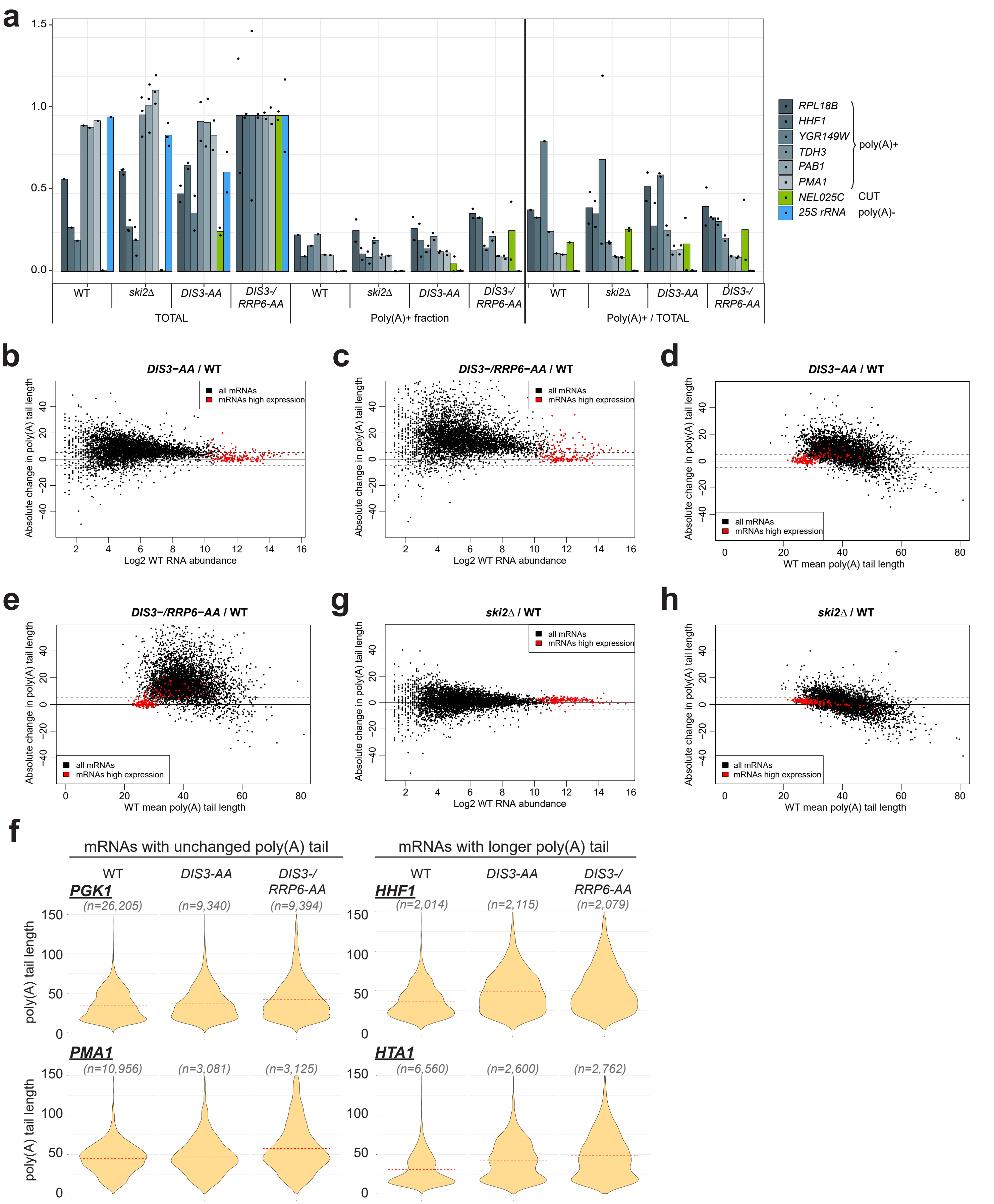

Supplemental Fig. 4 | a, Bar plots show in the leftmost panels internally normalized reverse-transcription qPCR-determined levels of selected RNAs in the total and poly(A)$^{+}$ fraction for WT, *DIS3-AA, DIS3-/RRP6-AA*, and *ski2Δ* strains. The rightmost panel shows the fraction of RNA that was recovered in the poly(A)$^{+}$ pool from the total. Dots depict single data points that produced the mean. As in Supplemental Fig. S1a, RNAs are grouped into poly(A)$^{+}$ mRNAs as gray series and poly(A)$^{-}$ as blue series. *NEL025c* CUT ncRNA is shown as a green series to represent exosome-dependent ncRNAs. **b, c,** Scatterplot of the relationship between log2-scaled mRNA abundance in WT cells and absolute change in mean poly(A)-tail length in *DIS3-AA* and *DIS3-/RRP6-AA* cells, respectively. Dashed lines mark a 5-adenosine change of mean poly(A)-tail length **d, e,** Scatterplot of the relationship between mean mRNA poly(A)-tail length in WT cells and absolute change in mean poly(A)-tail length in *DIS3-AA* and *DIS3-/RRP6-AA* cells, respectively. Dashed lines mark a 5-adenosine change of mean poly(A)-tail length **f,** Violin plots of the poly(A)-tail length distribution of selected mRNAs that had an unaffected or a strong increase in mean poly(A)-tail length upon Dis3 or Dis3 and Rrp6 depletion from the nucleus. **g, h,** Scatterplots showing the relationship between absolute change in mean poly(A)-tail length in *ski2Δ* cells (y-axis) and log2-scaled mRNA abundance in WT cells (g) or mean mRNA poly(A)-tail length in WT cells (h). Dashed lines designate a 5-adenosine change of mean poly(A)-tail length.

Supplemental Figure 5

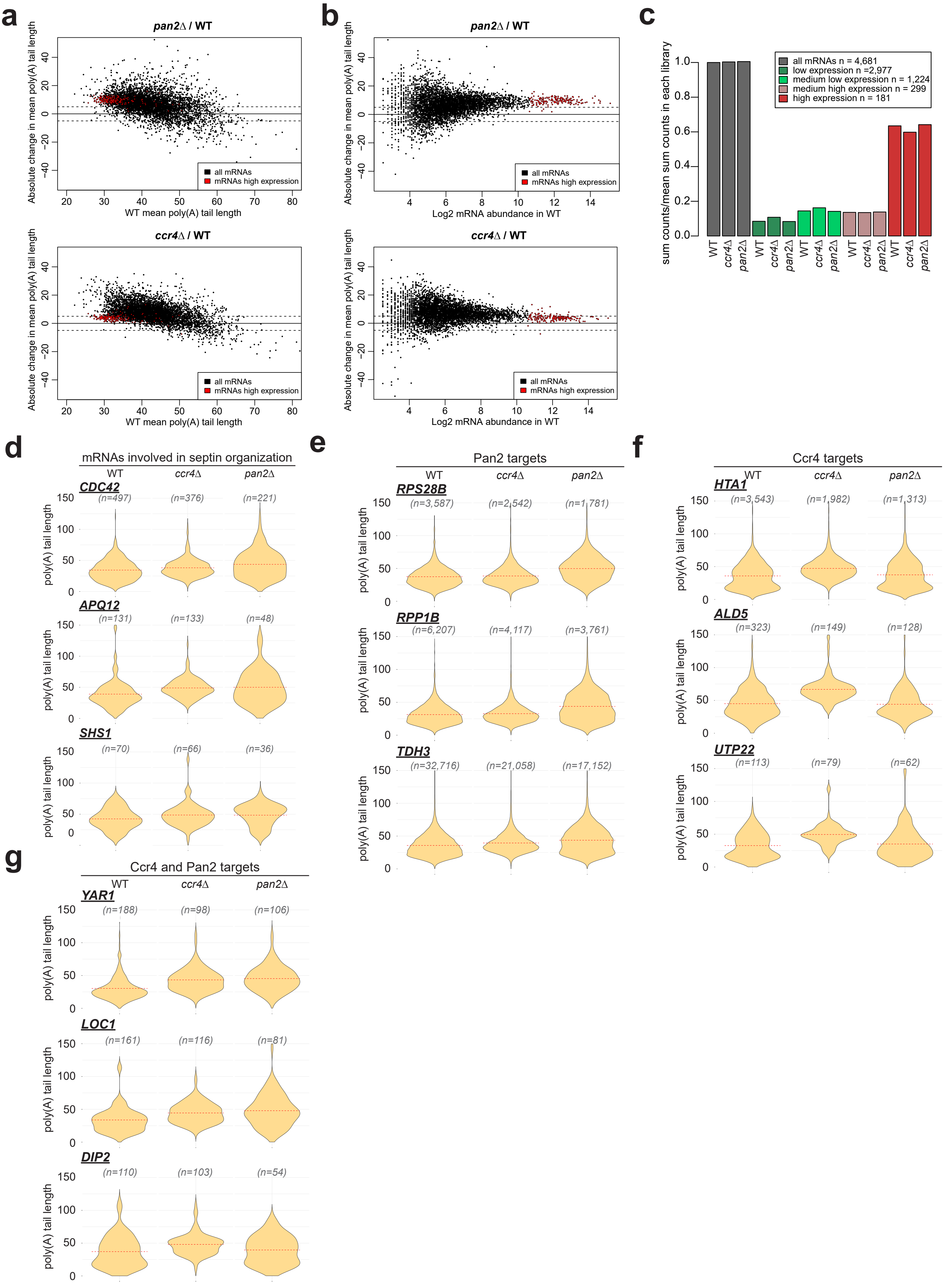

Supplemental Fig. 5 | a, b, Scatterplots of the relationship between absolute change in poly(A)-tail length for the *pan2Δ* and *ccr4Δ* strains (y-axis) and mean WT mRNA poly(A)-tail length (a) or log2-scaled mRNA abundance in WT cells (b). Dashed lines mark a 5-adenosine change of mean poly(A)-tail length. **c,** Barplot of the sum of counts (normalized to mean sum counts from each library) from mRNAs, grouped by expression level in WT. The key specifies the number of mRNAs in each category. **d,** Violin plots of the poly(A)-tail length distribution of DRS reads for three mRNAs involved in septin organization (reported by Traven et al., 2009[45]). **e – g,** Violin plots of the distribution of poly(A)-tail lengths of DRS-reads of selected transcripts identified as Pan2 targets (e), Ccr4 targets (f), or Ccr4 and Pan2 targets (g).

Supplemental Figure 6

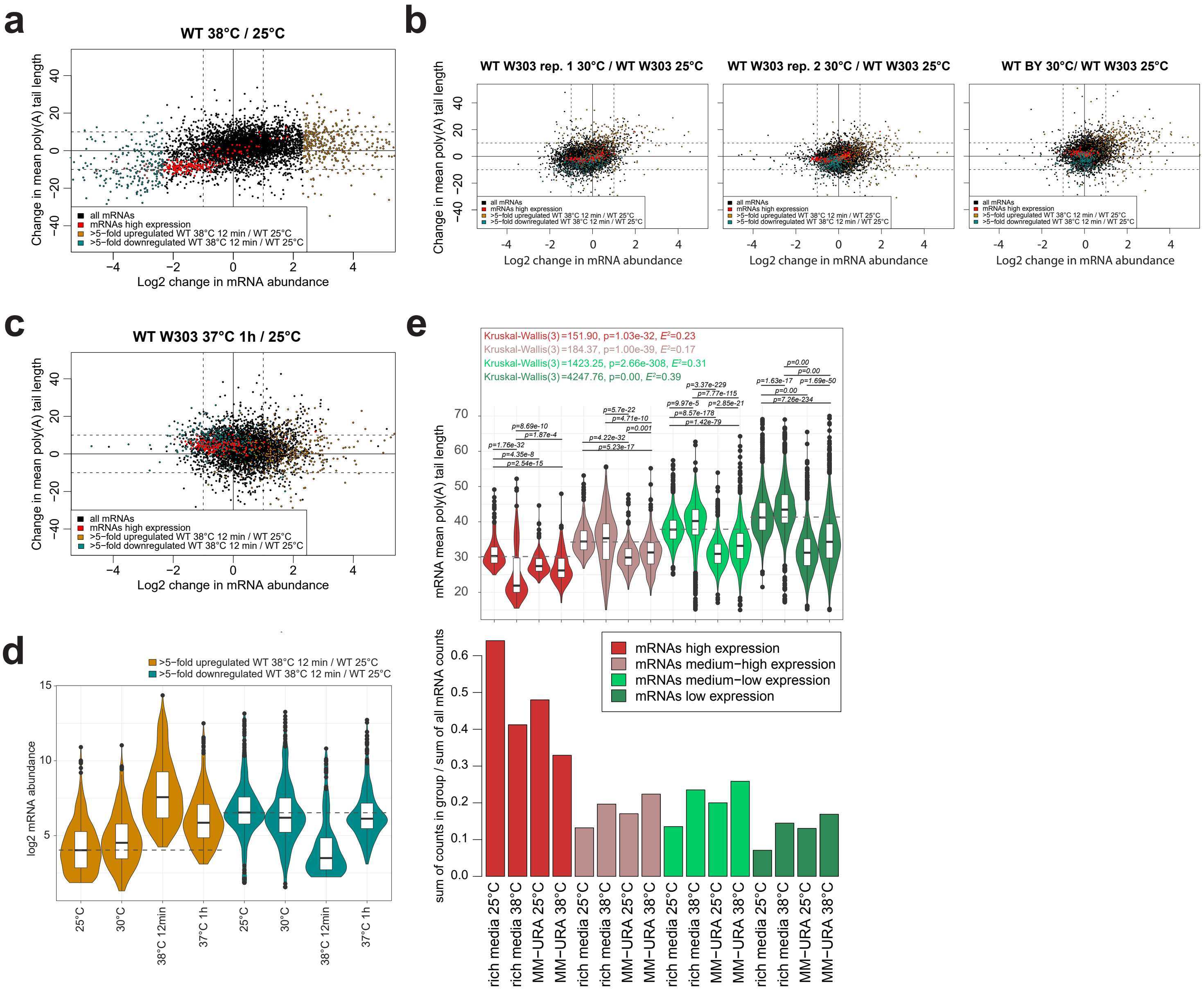

Supplemental Fig. 6 | a – c, Scatterplots related to Fig. 6a and b, showing the relationship between log2 fold-change in mRNA abundance (x-axis) and absolute change in poly(A)-tail length (y-axis) for cells that were grown at steady state at 25°C in rich media compared with brief heat shock (a), three replications of cells grown for 1 h at 30°C (b) and 37°C (c). Highly expressed mRNAs and mRNAs at least five-fold upregulated or down-regulated following heat-shock are marked in all graphs as indicated in the keys. Dashed lines designate a 2-fold change in expression and a 10-adenosine change of mean poly(A)-tail length **d,** Violin plot related to Fig. 6b shows log2 mRNA abundance of transcripts up- or down-regulated at least five-fold following heat shock in rich media. Only mRNAs detected in each growth condition are shown. **e,** The top panel shows the distribution of mean poly(A)-tail lengths of mRNAs, binned into four expression groups as in Fig. 6a. The bottom panel shows the sum of counts in each group, normalized to sum counts for all mRNAs. Only mRNAs that were detected in all four conditions (cells grown at steady state at 25°C in rich and MM-URA media compared with heat-stress at 38°C for 12 min) are included.

Supplemental Tables

Supplemental Table 1. List of yeast strains used in this study.

**Genotype**	**Source**	**Name**
BY4741; *MAT A; his3Δ1; leuΔ0; met15Δ0; ura3Δ0*	J. Kufel collection JW334	wild type BY
As BY4741; *rrp6Δ::NatMX6*	J. Kufel collection YW355 from Houseley Y01777	*rrp6Δ*
AS BY4741; *rrp6Δ::NatMX6; trf4Δ::KanMX4*	J. Kufel collection YW362 from LaCava et al., 2005	*rrp6Δ trf4Δ*
As BY4741; *trf4Δ::KanMX4*	J. Kufel collection YW356 from Euroscarf	*trf4Δ*
As BY4741; *trf5Δ::KanMX4*	J. Kufel collection YW357 form Euroscarf	*trf5Δ*
W303: *MAT A; leu2-3,112; trp1-1, can1-100; ura3-1, ade2-1; leu2-3,112; his3-11,15*	T.H. Jensen collection Y159	Wildtype W303
As W303; *MAT A; pap1-1*	T.H. Jensen collection Y2195	*pap1-1*
As W303; *MAT alpha; pap1-1; rrp6Δ::URA*	T.H. Jensen collection Y2196	*pap1-1 rrp6Δ*
As W303; *MAT alpha; pap1-1; trf4Δ::URA*	T.H. Jensen collection Y2197	*pap1-1 trf4Δ*
As W303; *MAT A; mft1Δ::KAN*	T.H. Jensen collection Y248	*mft1Δ*
As W303; *MAT alpha; tor1-1; fpr1::NAT; RPL13-2xFKBP12::TRP1; MEX67-FRB::kanMX6*; pRS426 (2μ, ori(f1), ori(pMB1), URA3, Ampr, LacZ, MCS, T7 promoter, T3 promoter)	T. H. Jensen collection Y2618 from Haruki et al., 2008, deposited at Euroscarf HHY182 Plasmid from pRS426 (ATCC 77107)	*Mex67-AA* pRS426
As W303; *MAT alpha; tor1-1; fpr1::NAT; RPL13-2xFKBP12::TRP1; DIS3-FRB::kanMX6*	T. H. Jensen collection Y3554	*Dis3-AA*
As W303; *MAT A; tor1-1; fpr1::NAT; RPL13-2xFKBP12::TRP1; DIS3-FRB::kanMX6; RRP6-FRB::kanMX6*	T. H. Jensen collection Y3590	*Dis3-AA Rrp6-AA*
As W303; *ski2Δ::HIS3*	T. H. Jensen collection Y242	*ski2Δ*
As W303; *MAT alpha CCR4::HIS3*	T. H. Jensen collection Y1900	*ccr4Δ*
As W303 *pan2Δ::HIS3*	T. H. Jensen collection Y313	*pan2Δ*

Supplemental Table 2. List of oligonucleotides used in this study.

**Name**	**Sequence**	**Source**
25S_fw	ATTCCCACTGTCCCTATCTACT	custom made Thermo Fisher
25S_rev	CTTGGCTGTGGTTTCGCT	custom made Thermo Fisher
CUT542_fw	ACTCTGCGATCCAAATTCTACT	custom made Thermo Fisher
CUT542_rev	ATGCGTCTTTCCTGTTTATGAG	custom made Thermo Fisher
DT18	TTTTTTTTTTTTTTTTTT	custom made Thermo Fisher
HHF1_qPCR_fw	ACTGCCCGGTTTTTCTTCT	custom made Thermo Fisher
HHF1_qPCR_rev	CCTAAACCCGCTATAATACACTCAT	custom made Thermo Fisher
Hsp104_3end_fwd	AGCTGAAGAATGTCTGGAAGT	custom made Thermo Fisher
Hsp104_3end_rev	CGTCATCACCTAACGTGTCA	custom made Thermo Fisher
LSR1_U2_FWD	AGCCATGACTGCATCTGTTG	custom made Thermo Fisher
LSR1_U2_REV	CTTTAAAAACAGGCGTCAACC	custom made Thermo Fisher
PAB1_YER165W_FWD	AAGACAAGCTTTGGGTGAAC	custom made Thermo Fisher
PAB1_YER165W_REV	CATCACTTTCCAACAATGGG	custom made Thermo Fisher
PMA1_middleA	TTGCCAGCTGTCGTTACCAC	custom made Thermo Fisher
PMA1_middleB	TCGACACCAGCCAAGGATTC	custom made Thermo Fisher
random hexamers	random hexamers	Invitrogen cat. no. 48190-011
RPL18B_FWD	CCACACAAGGGTAAGGCTCC	custom made Thermo Fisher
RPL18B_UTR_REV	GAAACAAGGAAAAAGGAACAAAG	custom made Thermo Fisher
RPL18B_FWD intron	AGCTGCCTATCGAGAATACC	custom made Thermo Fisher
RPL18B_REV2upst	GCTCTAGCAATTCTGGAGAC	custom made Thermo Fisher
RPL18B_probe	TTATACATGTATATATTGTAGTCAGTCATATGGTATGGAAAAATCCCTCTTT CCCAACCTAATATACACT	custom made Thermo Fisher
SCR1_fw	CCCGGCTATAATAAATCGATCT	custom made Thermo Fisher
SCR1_rev	GCTGACGCTGGATAAAACT	custom made Thermo Fisher
snR13 probe	CAACTCGAGCCAAATGCACTC	custom made Thermo Fisher
SSA4_fwd	AAATTGTACTTGTTGGTGGTTCA	custom made Thermo Fisher
SSA4_rev	GGGTTAATCGAACGGTTTGG	custom made Thermo Fisher
TDH3_fw	CTCTCACTCTTCCATCTTCGAT	custom made Thermo Fisher
TDH3_rev	CGTACCAGGAGACCAACTT	custom made Thermo Fisher
TDH3_probe	AGCCTTGGCAACGTGTTCAACCAAGTCGACAACTCTGGTAGAGTAACCG TATTCGTTGTCG	custom made Thermo Fisher
YGR149W_FWD	ATGGGCGTCTCATAATGGGG	custom made Thermo Fisher
YGR149W_REV	CACGGAGGCATCAGATATGA	custom made Thermo Fisher
RLucF1	gccatcagattgtgtttgttagtcgctATGATTCCGAGAAGCACGCCGAGAAC	custom made Thermo Fisher
RLucR1	*gcttacggttcactactcacgacgatg*TTACTGCTCGTTCTTCAGCACGCG	custom made Thermo Fisher
RLuc_T7_F2	TAATACGACTCACTATAGGGAGAgccatcagattgtgtttgttagtcgct	custom made Thermo Fisher
RLuc_A10_R2	TTTTTTTTTT*gcttacggttcactactcacgacgatg*	custom made Thermo Fisher
RLuc_A15_R2	TTTTTTTTTTTTTTT*gcttacggttcactactcacgacgatg*	custom made Thermo Fisher
RLuc_A30_R2	TTTTTTTTTTTTTTTTTTTTTTTTTTTTTT*gcttacggttcactactcacgacgatg*	custom made Thermo Fisher
RLuc_A45_R2	TTTTTTTTTTTTTTTTTTTTTTTTTTTTTTTTTTTTTTTTTTTTT*gcttacggttcact actcacgacgatg*	custom made Thermo Fisher
RLuc_A60_R2	TTTTTTTTTTTTTTTTTTTTTTTTTTTTTTTTTTTTTTTTTTTTTTTTTTTTTTT TTTTT*gcttacggttcactactcacgacgatg*	custom made Thermo Fisher
RLuc_A90_R2	TTTTTTTTTTTTTTTTTTTTTTTTTTTTTTTTTTTTTTTTTTTTTTTTTTTTTTT TTTTTTTTTTTTTTTTTTTTTTTTTTTTTTTTTTT*gcttacggttcactactcacgacgatg*	custom made Thermo Fisher

Supplemental Table 3. List of deposited DRS datasets. The WT or Mex67-AA -rapamycin samples are controls to mutant/depletion strains that follow. Unless specified otherwise, sequencing was performed on polyA^{+} fraction.

**Sample**	**Accession number at ENA**
DRS WT BY4741 30°C control to *rrp6Δ*, *trf4Δ*, *trf5Δ* and *rrp6Δ trf4Δ*	ERS4936519, ERS3526067, ERS4936515
DRS *rrp6Δ* 30°C	ERS4936517, ERS5470421, ERS5470422
DRS *trf4Δ* 30°C	ERS5465275, ERS4936520, ERS5464585
DRS *trf5Δ* 30°C	ERS5465276, ERS4936521, ERS5464586
DRS *rrp6Δ trf4Δ* 30°C	ERS5458723, ERS5458724
DRS WT W303 30°C<br>control to *pap1-1, DIS3-AA* series and *ski2Δ*	ERS5458726, ERS5458727
DRS *pap1-1* W303 30°C	ERS5458725, ERS5458728
DRS *pap1-1 rrp6Δ* W303 30°C	ERS4936516, ERS4936518
DRS *pap1-1 trf4Δ* W303 30°C	ERS4936513, ERS4936512
DRS *DIS3-AA* W303 30°C	ERS5464062, ERS5464063
DRS *DIS3-AA RRP6-AA* W303 30°C	ERS5464064, ERS5464065
DRS *ski2Δ* W303 30°C	ERS5458721, ERS5458722
DRS WT W303 30°C<br>control to deadenylase series	ERS5464066, ERS5464067
DRS *ccr4Δ* W303 30°C	ERS5084525, ERS5084526
DRS *pan2Δ* W303 30°C	ERS5084529, ERS5084530
DRS WT W303 25°C control to *mft1Δ* 25°C and 38°C 12 min and WT 38°C 12 min	ERS5465444, ERS5465445
DRS *mft1Δ* W303 25°C	ERS5465446, ERS5465447, ERS5470413,ERS5470414
DRS WT W303 38°C 12 min	ERS5465448, ERS3526064
DRS *mft1Δ* W303 38°C 12 min	ERS5465450, ERS5465451
DRS *MEX67-AA* – rapamycin W303 25 °C MM-URA	ERS5465452, ERS5465453, ERS5470417, ERS5470416
DRS *MEX67-AA* – rapamycin W303 38 °C 15 min MM-URA	ERS5465454, ERS5465455
DRS *MEX67-AA* + rapamycin W303 38 °C 15 min MM-URA	ERS5465456, ERS5465457
DRS WT W303 37°C 60 min	ERS5470037, ERS5470039
DRS on total RNA WT W303 30°C (control RNA for *ccr4Δ* and *pan2Δ*)	ERS6477293, ERS6477294, ERS6477295
Poly(A) standards (10A, 15A, 30A, 45A, 60A, 90A)	ERS6477287, ERS6477288, ERS6477289, ERS6477290, ERS6477291, ERS6477292

Supplemental References

1. Presnyak, V. *et al.* Codon Optimality Is a Major Determinant of mRNA Stability. *Cell* **160**, 1111–1124 (2015).
2. Gudipati, R. K. *et al.* Extensive Degradation of RNA Precursors by the Exosome in Wild-Type Cells. *Mol. Cell* **48**, 409–421 (2012).
3. Subtelny, A. O., Eichhorn, S. W., Chen, G. R., Sive, H. & Bartel, D. P. Poly(A)-tail profiling reveals an embryonic switch in translational control. *Nature* **508**, 66–71 (2014).
4. Tudek, A. *et al.* A Nuclear Export Block Triggers the Decay of Newly Synthesized Polyadenylated RNA. *Cell Rep.* **24**, 2457-2467.e7 (2018).
5. Schmid, M., Tudek, A. & Jensen, T. H. Simultaneous Measurement of Transcriptional and Post-transcriptional Parameters by 3′ End RNA-Seq. *Cell Rep.* **24**, 2468-2478.e4 (2018).
6. Pelechano, V., Chávez, S. & Pérez-Ortín, J. E. A Complete Set of Nascent Transcription Rates for Yeast Genes. *PLOS ONE* **5**, e15442 (2010).
7. Neymotin, B., Athanasiadou, R. & Gresham, D. Determination of in vivo RNA kinetics using RATE-seq. *RNA* **20**, 1645–1652 (2014).
