# Supplementary material for: Global view on the metabolism of RNA poly(A) tails in yeast Saccharomyces cerevisiae
Source: Nat Commun. 2021 Aug 16;12:4951. doi: 10.1038/s41467-021-25251-w (PMC8367983; doi:10.1038/s41467-021-25251-w)
Supplement: Supplementary file 5 — Description of additional supplementary files [file 41467_2021_25251_MOESM5_ESM.docx]

Description of additional supplementary files

Title: Supplementary Data 1

Description: List of mRNAs up-/down-regulated following heatstress in rich media and list of mRNAs with a large oligo-adenylated fraction

Title: Supplementary Data 2

Description: List of sequencing runs included in the study
